# Evaluating the Use of Anxiety Patient‐Reported Outcome Measures (PROMs) in Dementia Clinical Trials: A Systematic Review

**DOI:** 10.1111/hex.70736

**Published:** 2026-06-21

**Authors:** Jason Domingo, Ana Alves, Julie Sanders

**Affiliations:** ^1^ National Institute for Health and Care Research—Cambridge Clinical Research Facility Cambridge University Hospitals NHS Foundation Trust Cambridge UK; ^2^ Guy's and St Thomas’ NHS Foundation Trust London UK; ^3^ Faculty of Nursing, Midwifery & Palliative Care King's College London London UK

**Keywords:** Alzheimer's disease, anxiety, dementia, patient‐reported outcome measures (PROMs), randomised controlled trials (RCT), systematic review

## Abstract

Anxiety is a common but underdetected or underdiagnosed symptom in dementia, affecting quality of life and care outcomes. Clinical trials are essential for informing effective management, yet the use of patient‐reported outcome measures (PROMs) in dementia trials remains unclear. This review examined how validated PROMs are used to assess anxiety in dementia trials, including measurement tools, methods, demographic representation and evidence gaps.

**Methods:**

A systematic review was conducted following PRISMA and Cochrane Handbook guidance, with protocol registration on PROSPERO (CRD42025649920). Randomised controlled trials published between January 2015 and February 2025 were included if they assessed anxiety in mild to moderate dementia using validated PROMs within pharmacological or non‐pharmacological interventions. Searches were conducted across MEDLINE, Embase, PsycINFO, Cochrane Library and ClinicalTrials.gov, supplemented by citation tracking. Two reviewers independently undertook study selection, data extraction and risk of bias assessment. Due to heterogeneity, findings were narratively synthesised.

**Results:**

Of 2328 records screened, 29 trials (*n* = 5697) met inclusion criteria. While 93.1% used validated anxiety measurement tools, only 44.4% employed PROMs, most frequently the Hospital Anxiety and Depression Scale, mainly in non‐pharmacological trials. Women and individuals of White ethnicity were overrepresented, and no studies examined PROM effectiveness by sex and ethnicity. Most trials showed moderate to high risk of bias, and evidence was confined to high‐income countries.

**Conclusions:**

Anxiety outcomes in dementia trials remain largely proxy‐reported. Existing anxiety PROMs are generic and unvalidated for dementia populations. There is a critical need for dementia‐specific, culturally sensitive anxiety PROMs, improved demographic reporting and integration of anxiety assessment within dementia core outcome sets.

**Patient or Public Contribution:**

Feedback from research participants in dementia studies, who reported experiencing anxiety, motivated this systematic review. Patient and public involvement was examined across all included studies. The lack of validated dementia‐specific anxiety PROMs identified in this review highlights a broader gap in co‐produced outcome development within dementia research. These findings emphasise the need for meaningful involvement of people living with dementia and care partners in the development, validation and cultural adaptation of anxiety PROMs to ensure that trial outcomes are relevant, acceptable and representative.

## 1 Background

Dementia is a progressive neurodegenerative syndrome characterised by decline across multiple cognitive domains including attention, memory, language, executive functioning, perceptual‐motor ability and social cognition, which collectively impair an individual's ability to maintain independence in daily life [1]. Globally, approximately 55 million people lived with dementia in 2020, a figure projected to increase by 41.82% by 2030 and 152.73% by 2050 [2], with nearly 60% of cases residing in low‐ and middle‐income countries [3]. In the United Kingdom, about 982,000 people are currently affected, with prevalence expected to rise by 40% by 2040 [4]. Despite higher risk, women and racial or ethnic minority groups remain underrepresented in dementia research, limiting understanding of subgroup‐specific risk factors and intervention responses [5, 6]. Dementia comprises several subtypes including Alzheimer's disease, vascular dementia, dementia with Lewy bodies and frontotemporal dementia, each with distinct causes, symptom profiles and progression rates [7, 8]. There is currently no treatment capable of slowing or halting disease progression.

Anxiety is a highly prevalent neuropsychiatric symptom in dementia, affecting between 40% and 71% of individuals depending on dementia subtype and patient characteristics [9]. Although anxiety is recognised as a component of behavioural and psychological symptoms of dementia (BPSD), it may be underdetected or underdiagnosed within broader neuropsychiatric assessments [10]. Symptoms include psychological features such as fear, restlessness, heightened emotional sensitivity, difficulty concentrating and irritability, and alongside physical manifestations including dizziness, sleep disturbances, palpitations, headaches, gastrointestinal distress, sweating and shortness of breath [1]. As dementia progresses, anxiety symptoms often intensify in parallel with cognitive and functional decline, which complicates diagnosis and management [11]. Maintaining routine and a stable environment is central to reducing anxiety, while disruptions, including clinical trial participation, may exacerbate symptoms [9, 12]. Anxiety assessment in dementia commonly involves clinician‐reported outcomes (ClinRO), observer‐reported outcomes (ObsRO) completed by caregivers, and patient‐reported outcome measures (PROMs), which directly capture the patient's perspective [13] and support patient autonomy [14].

PROMs are standardised and validated tools that collect information directly from patients regarding their functional health status or quality of life without external interpretation [15]. They are widely used in clinical decision‐making, quality improvement, benchmarking performance, policy development and patient–clinician communication. PROMs may be generic, such as the EQ‐5D [16], or condition‐specific, such as DEMQOL, QOL‐AD, BASQID, DQoL, ADRQL and QUALIDEM for dementia [17, 18]. Several validated anxiety PROMs exist, including GAD‐7 [19, 20], HADS [14], BAI [21], STAI [22], GAS [23] and SAST [24], but their frequency and appropriateness in dementia trials remain unclear [14, 19]. While several dementia‐specific PROMs exist, their integration into dementia registries remains limited, reflecting challenges in standardised implementation across clinical and research settings. Their use in research is also shaped by the progressive nature of dementia, as declining cognitive and communicative capacity limits the feasibility of self‐report in advanced stages of the disease; consequently, proxy‐reported outcomes are commonly used to ensure that symptom data can still be reliably captured [18]. Anxiety is also omitted from existing core outcome sets [25], suggesting that psychological and emotional dimensions may have been overlooked in consensus‐driven outcome frameworks. This literature review evaluates how anxiety is measured in dementia trials, the instruments used and whether PROM effectiveness varies by sex or ethnicity.

## 2 Methods

This systematic review was registered with PROSPERO, an international prospective register of systematic reviews (reference number CRD42025649920; February 2025) [26], and is reported in accordance with the Preferred Reporting Items for Systematic Reviews and Meta‐Analyses (PRISMA) [27, 28] and Cochrane Handbook guidelines [29].

### 2.1 Eligibility Criteria

The inclusion and exclusion criteria are summarised in Table 1. Studies were eligible if they involved individuals diagnosed with mild to moderate dementia, confirmed using validated rating scales such as the Clinical Dementia Rating (CDR) Scale, Mini‐Mental State Examination (MMSE) or the Global Deterioration Scale (GDS). This population was selected because individuals with mild to moderate dementia are more likely to retain sufficient cognitive capacity to understand, reflect on and self‐report symptoms, which is a key requirement for PROMs. In contrast, individuals with severe or late‐stage dementia often experience significant cognitive and communicative impairments that limit the reliability and feasibility of self‐report, making proxy‐reported outcomes a more appropriate and commonly used approach in this stage of the disease. Only randomised controlled trials assessing pharmacological or non‐pharmacological interventions in Alzheimer's dementia, vascular dementia, dementia with Lewy bodies, or frontotemporal dementia were included. Studies published in English between January 2015 and February 2025 were included to provide a contemporary overview of anxiety assessment practices and the use of PROMs in dementia trials over the past decade. Exclusion criteria comprised non‐human, observational or non‐clinical studies, and studies focusing on non‐dementia conditions, and those involving late‐stage or severe dementia.

**Table 1:** Eligibility criteria.

Inclusion criteria		Exclusion criteria
Study design	Randomised controlled trials involving pharmacological and non‐pharmacological interventions for dementia	Non‐human studies
		Non‐dementia related studies
		Observational or non‐clinical trials
		Studies focusing on late‐stage or severe type of dementia as confirmed by validated rating scales such as the Clinical Dementia Rating (CDR) Scale, Mini‐Mental State Examination (MMSE), or the Global Deterioration Scale (GDS)
Dementia types *based on International Classification of Diseases (ICD‐10)*	Alzheimer's dementia	
	Vascular dementia	
	Dementia with Lewy bodies	
	Frontotemporal dementia	
Participant characteristics	Early to middle stages of dementia as confirmed by validated rating scales such as the Clinical Dementia Rating (CDR) Scale, Mini‐Mental State Examination (MMSE), or the Global Deterioration Scale (GDS)	
	No age restrictions	
Language	Studies published in English	
Publication date range	Studies published from January 2015 to February 2025	

### 2.2 Information Sources, Search Strategy and Study Selection

A systematic search was conducted across five electronic databases: MEDLINE (Ovid), Embase, the Cochrane Library (for systematic reviews and trials), PsycINFO and ClinicalTrials.gov. Relevant articles published in English between January 2015 and February 2025 were considered. In addition, backward citation searching was undertaken through manual screening of reference lists and citations of all included studies that met the eligibility criteria.

To optimise the search strategy, truncation and wildcard symbols (‘*’, ‘#’), and Boolean operators (OR, AND, NOT) were applied to combine controlled vocabulary terms and free‐text keywords when searching the titles and abstracts of relevant studies. The finalised MEDLINE (Ovid) search strategy was translated for use in other databases using their respective controlled vocabularies (Supporting Information). To ensure methodological rigour, a Specialist Clinical Librarian from Cambridge University Hospitals NHS Foundation Trust reviewed the MEDLINE (Ovid) search strategy and provided guidance on its translation to other platforms.

Two authors independently screened titles and abstracts for suitability against the predefined inclusion and exclusion criteria (Table 1) using Zotero software version 7.0.15 (16 March 2025) and Rayyan 2025 software [30]. Full‐text articles deemed potentially relevant were subsequently assessed for inclusion. Disagreements during the screening process were resolved through discussion and consensus.

### 2.3 Data Extraction and Synthesis

The lead author conducted full data extraction, whilst a second author independently verified a random 10% sample (*n* = 3) of the extracted data using a bespoke proforma. Any discrepancies were resolved through discussion until consensus was achieved.

Extracted data included author, year of publication, country, sample size (enrolled and completed), study setting, participant demographics (mean age, dementia type and severity, sex, and race), patient and public involvement (PPI) in PROM used (development and/or validation), treatment categories, total study duration and details of anxiety assessment (if conducted), including assessment frequency, timepoints and tools utilised (PROMs or non‐PROMs).

### 2.4 Risk of Bias and Quality Assessment

Study quality and risk of bias were assessed using the Critical Appraisal Skills Programme (CASP) Checklist for Randomised Controlled Trials (RCTs) [31, 32, 33] and the revised Cochrane Risk of Bias tool for randomised trials (RoB 2.0) [29]. A secondary reviewer independently verified 10% of the included studies. Any disagreements were resolved through discussion until consensus was reached.

### 2.5 Analysis

Results were synthesised using tables and narrative summaries as appropriate. Interpretation of the findings was discussed and agreed upon by all members of the authorship team. Meta‐analysis was not conducted due to substantial heterogeneity across studies.

## 3 Results

**Figure 1:**
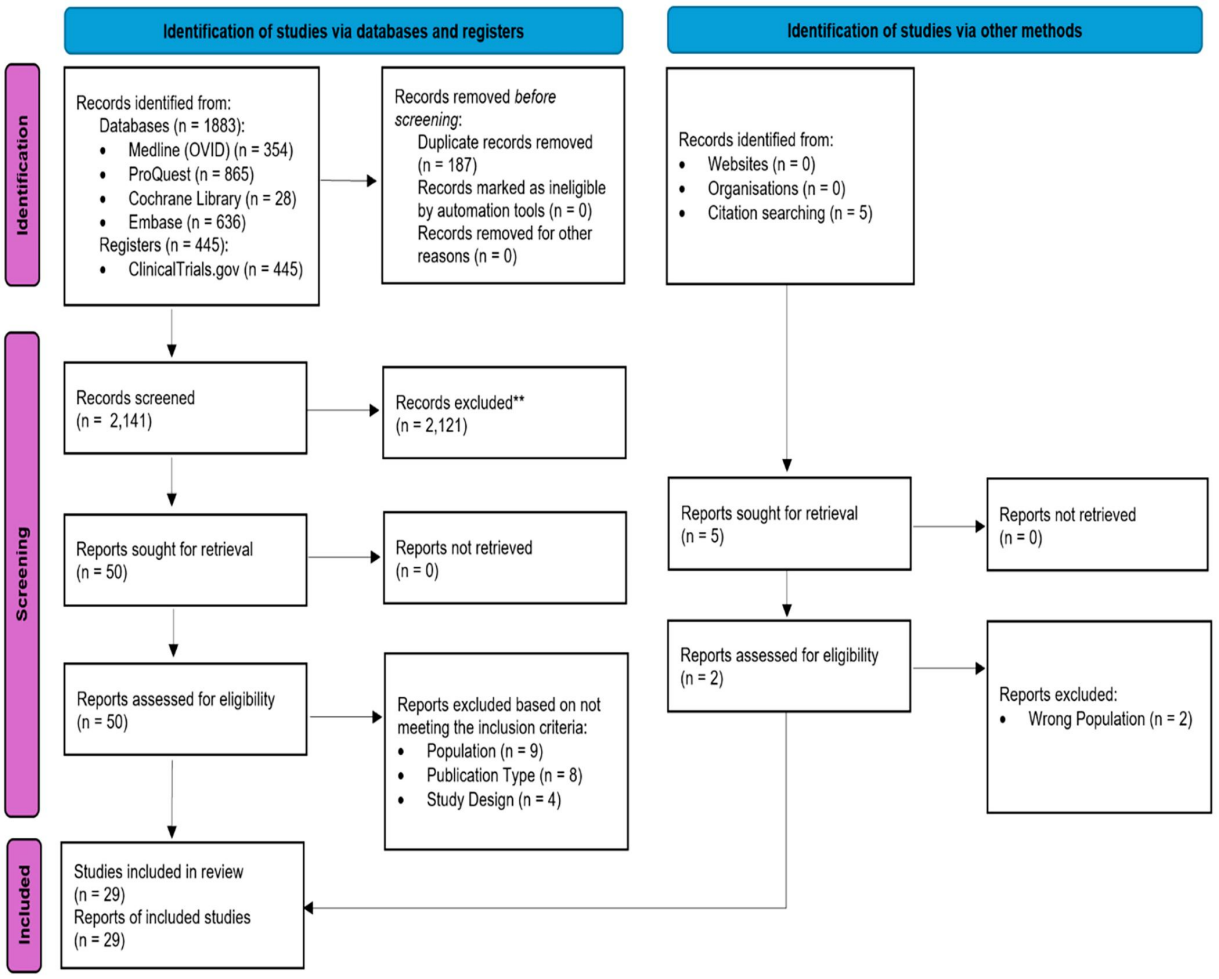
PRISMA flow diagram.

A total of 2328 records were identified through electronic database searches (Figure 1), with 50 full‐text articles independently assessed for eligibility. This resulted in 29 papers being included for data synthesis.

### 3.1 Study Characteristics

The 29 included studies comprised a total of 5697 participants with dementia, with a pooled mean age of 75.7 years (Table 2). Most studies were conducted in Europe, the United States and the United Kingdom (*n* = 17, 58.62%), with the remainder in Asia (*n* = 12, 41.38%). The majority were multi‐centre, though single‐centre studies were more common in Asia.

**Table 2:** Study characteristics.

Study (Author, Year)	Trial location		Study duration (months)	Population			
	Country	Number of sites (*n*)		Mean age (years)	Sample size Enrolled (*n*) Completed (*n*) (%)	Type of dementia	Severity of dementia
Spector et al. [34]	United Kingdom	Multi‐centre (2)	6	78.5	50 38 (76.00%)	Alzheimer's disease	Mild to moderate
Henderson et al. [35]	United States of America	Multi‐centre (4)	12	76.0	42 39 (92.86%)	Alzheimer's disease	Mild to moderate
Quinn et al. [36]	United Kingdom	Single centre (1)	6	75.7	24 23 (95.83%)	Alzheimer's disease Vascular dementia	Mild
Nacu et al. [37]	Moldova and Belarus	Multi‐centre (not specified)	6	65.0	410 402 (98.05%)	Alzheimer's Disease Vascular dementia	Mild to moderate
Pongan et al. [38]	France	Multi‐centre (3)	4	79.5	65 59 (90.77%)	Alzheimer's disease	Mild
Cheung et al. [39]	Hong Kong	Multi‐centre (12)	3	85.3	165 124 (75.15%)	Alzheimer's disease	Moderate
Egan et al. [40]	United States of America, Europe, Australia, New Zealand, Japan and others	Multi‐centre (238)	20	71.8	1958 1389 (70.94%)	Alzheimer's disease	Mild to moderate
Clare et al., 2019 [41]	United Kingdom	Multi‐centre (8)	6	78.6	474 426 (89.87%)	Alzheimer's disease Vascular dementia	Mild
Zhou et al. [42]	China	Single centre (1)	3	71	80 78 (97.5%)	Alzheimer's disease	Moderate
Koch et al. [43]	Italy	Multi‐centre (not specified)	6	73.9	94 78 (82.98%)	Alzheimer's disease	Mild to moderate
Maier et al. [44]	Germany	Multi‐centre (not specified)	3	74.8	110 81 (73.64%)	Alzheimer's disease	Mild
Li et al. [45]	China	Single centre (1)	6	83.4	90 85 (94.44%)	Alzheimer's disease	Mild to moderate
Jenewein et al. [46]	Switzerland	Single centre (1)	3	79.6	54 48 (88.89%)	Alzheimer's disease	Mild
Tonga et al. [47]	Norway	Multi‐centre (5)	10	70.1	198 131 (66.17%)	Alzheimer's disease	Mild
Banerjee et al. [48]	United Kingdom	Multi‐centre (26)	3	82.5	204 171 (83.82%)	Alzheimer's disease	Mild
Liu et al. [49]	Taiwan	Multi‐centre (not specified)	3	86.8	50 50 (100.00%)	Alzheimer's disease	Mild to moderate
Akhgarjand et al. [50]	Iran	Multi‐centre (not specified)	3	67.0	90 90 (100.00%)	Alzheimer's disease	Mild to moderate
Wu et al. [51]	China	Single centre (1)	2.5	66.0	49 47 (95.92%)	Alzheimer's disease	Mild
Menengi et al. [52]	Turkey	Single centre (1)	1.5	79.2	20 20 (100.00%)	Alzheimer's disease	Mild to moderate
Bhowmik et al. [53]	India	Multi‐centre (22),	6	67.2	100 57 (57.00%)	Alzheimer's disease Vascular dementia Fronto‐temporal Lewy Bodies (LBD)	Mild to moderate
Monteiro et al. [54]	United States of America, Europe: France, Poland, Spain	Multi‐centre (49)	12	72.0	272 208 (76.47%)	Alzheimer's disease	Mild to moderate
Jung et al. [55]	Korea	Multi‐centre (2)	2	77.4	52 42 (80.77%)	Alzheimer's disease	Mild to moderate
Atay et al. [56]	Turkey	Single centre (1)	3	74.9	52 52 (100.00%)	Alzheimer's disease	Mild
Howard et al. [57]	United Kingdom	Multi‐centre (24)	12	77.0	336 279 (83.04%)	Alzheimer's disease Vascular dementia	Mild to moderate
Christakou et al. [58]	Greece	Multi‐centre (2)	3	77.5	160 142 (88.75%)	Alzheimer's disease	Mild
Maleki et al. [59]	Iran	Single centre (1)	3	74.2	54 53 (98.15%)	Alzheimer's disease	Mild to moderate
Fleisher et al. [60]	Canada, Japan, United States of America	Multi‐centre (56)	25	75.4	360 218 (60.56%)	Alzheimer's disease	Mild
Forstmeier et al. [61]	Switzerland	Multi‐centre (2)	12	75.5	41 24 (58.44%)	Alzheimer's disease Vascular dementia	Mild
Dengiz et al. [62]	Turkey	Single centre (1)	1.5	79.3	43 37 (86.05%)	Alzheimer's disease	Mild

Sample sizes were relatively small, ranging from 24 to 200 participants. Of those enrolled, 4521 participants (79.36%) completed the studies. Intervention duration varied across studies, with most lasting 3–10 months. Most studies (*n* = 23, 79.31%) focused exclusively on Alzheimer's Disease, with fewer including vascular, frontotemporal, or Lewy body dementias. Nearly half of the studies (*n* = 14, 48.28%) included participants with mild dementia only, with the remainder including both mild and moderate stages.

### 3.2 Risk of Bias

**Figure 2:**
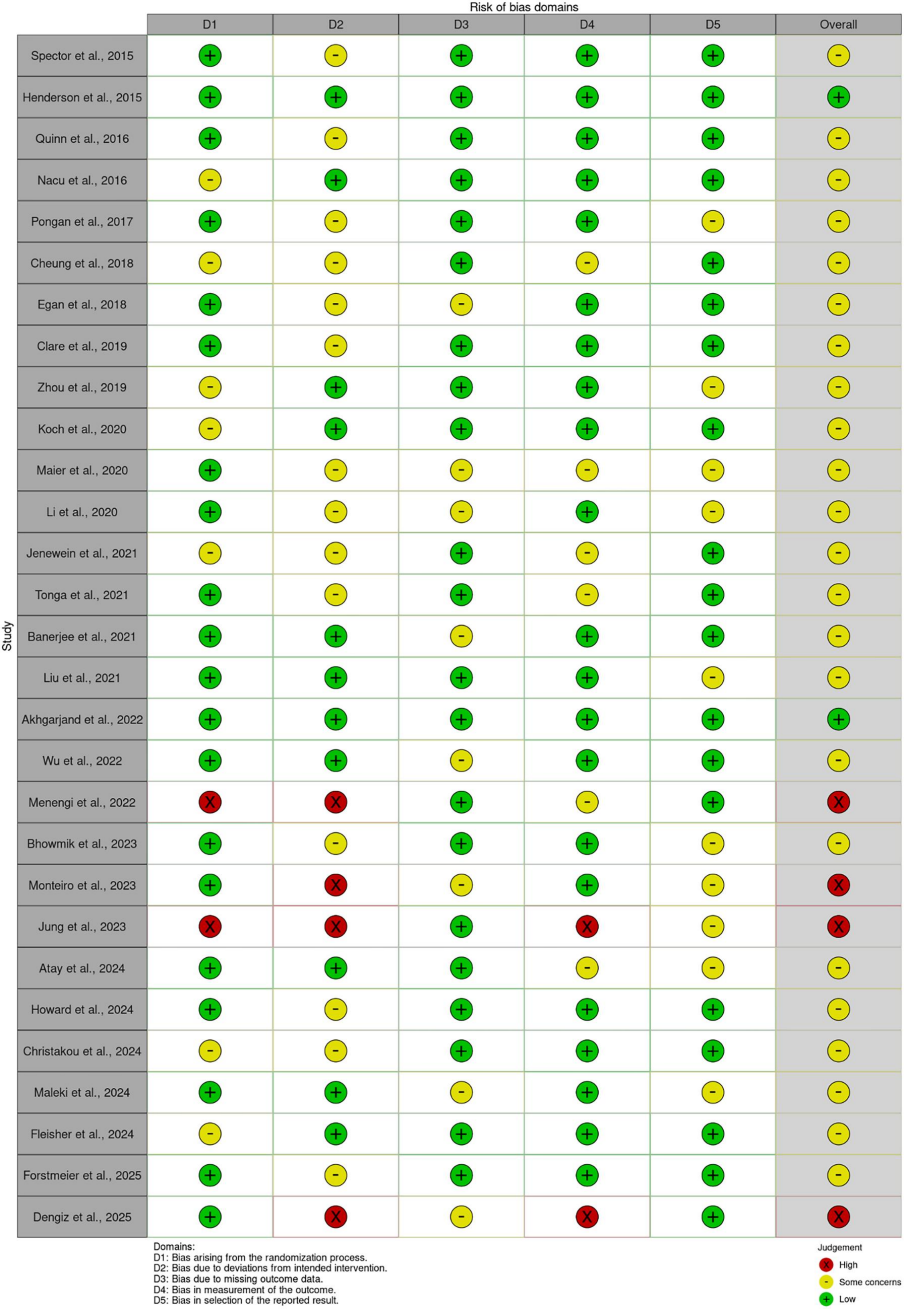
Risk of bias for individual studies.

**Figure 3:**
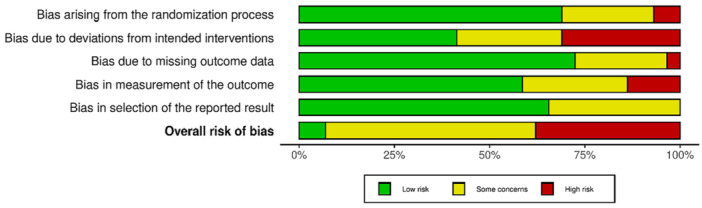
Risk of bias summary: Risk of bias item presented as percentages across all included studies.

Overall, most studies were assessed as having some concerns or a moderate risk of bias, with only a small proportion rated as low risk and a minority as high risk. Across domains, randomisation procedures were generally adequate, while concerns most commonly arose from deviations from intended interventions and incomplete reporting of blinding. Missing outcome data and outcome measurement were typically judged as low risk, although unclear handling of missing data and lack of assessor blinding were recurrent issues. Figures 2 and 3 illustrate the variability in risk of bias across individual studies and by domain.

### 3.3 Anxiety Assessment and Measurement

Treatment allocation and anxiety assessment characteristics across the included studies are summarised in Table 3. Among the 29 studies, anxiety was assessed in 27 studies (93.1%) either directly or indirectly as a primary (*n* = 10, 34.48%) or secondary (*n* = 17, 58.62%) outcome. The remaining two studies (6.90%) did not formally measure anxiety but identified anxiety‐related symptoms as adverse events or treatment‐emergent adverse events.

**Table 3:** Anxiety assessment and measurement.

Study (Author, Year)	Treatment allocation			Anxiety assessment			Anxiety measurement		Patient and public involvement (PPI) Yes = Y No = N
	Treatment and number of participants allocated (*n*)	Comparator and number of participants allocated (*n*)	Category of treatment modality P = Pharmacological NP = Non‐pharmacological	**Anxiety Assessed** Yes = Y (Primary/Secondary Outcome) No = N	Frequency (*n*)	Time points	Tools used to measure anxiety	**PROMs used Yes = Y No = N**	
Spector et al. [34]	Cognitive behavioural treatment and treatment as usual (25)	Treatment as usual alone (25)	NP	Y (Primary)	3	Baseline, week 15, month 6	Hospital Anxiety and Depression Scale (HADS)	Y	N
Henderson et al. [35]	Oral raloxifene hydrochloride (21)	Placebo (21)	P	Y (Secondary)	3	Baseline, month 6, month 12	Neuropsychiatric Inventory (NPI)	N	N
Quinn et al. [36]	Self‐management group (13)	Treatment as usual (11)	NP	Y (Secondary)	3	Baseline, month 3, month 6	Hospital Anxiety and Depression Scale (HADS)	Y	N
Nacu et al. [37]	Ginkgo Biloba Extract EGb 761 (205)	Placebo (205)	P	Y (Primary)	2	Baseline, week 24	Neuropsychiatric Inventory (NPI)	N	N
Pongan et al. [38]	Choral singing intervention (31)	Painting session intervention (28)	NP	Y (Secondary)	3	Baseline, week 12, week 16	State Trait Anxiety Inventory (STAI)	Y	N
Cheung et al. [39]	Music ‐ with ‐ movement (58)	‐ Music preferred listening (54)‐ social activity group or chatting (53)	NP	Y (Primary)	3	Baseline, week 6, week 6 post	Rating Anxiety in Dementia (RAID)	N	N
Egan et al. [40]	Oral 12 mg verubecestat dose (653)	‐ Oral 40 mg verubecestat dose (652)‐ placebo (653)	P	Y (Secondary)	2	Baseline, month 20	Neuropsychiatric Inventory (NPI)	N	N
Clare et al. [41]	Individualised goal‐oriented cognitive rehabilitation session (238)	Treatment as usual (236)	NP	Y (Secondary)	3	Baseline, month 3, month 6	Hospital Anxiety and Depression Scale (HADS)	Y	Y
Zhou et al. [42]	Oral memantine and citalopram (40)	Oral memantine and placebo (40)	P	Y (Primary)	2	Baseline, month 3	Neuropsychiatric Inventory (NPI)	N	N
Koch et al. [43]	Rotigotine transdermal patch (47)	Placebo transdermal patch (47)	P	Y (Secondary)	2	Baseline, week 24	Neuropsychiatric Inventory (NPI)	N	N
Maier et al. [44]	Oral bupropion on titrated dose (55)	Placebo (55)	P	Y (Secondary)	4	Baseline, week 4, week 8, week 12	Quality of Life in Alzheimer's Disease Scale (QoL‐AD)	N	N
Li et al. [45]	Group‐based reminiscence therapy and conventional treatment (45)	Conventional drug treatments and routine daily care (45)	NP	Y (Secondary)	4	Baseline, week 4, week 12, week 24	Neuropsychiatric Inventory (NPI)	N	N
Jenewein et al. [46]	Immediate dignity therapy (28)	Delayed dignity therapy (26)	NP	Y (Secondary)	3	Baseline, week 1, month 3	Hospital Anxiety and Depression Scale (HADS)	Y	N
Tonga et al. [47]	Multi‐component psychotherapy interventions (100)	Treatment as usual (98)	NP	Y (Secondary)	3	Baseline, month 4, month 10	Hospital Anxiety and Depression Scale (HADS)	Y	N
Banerjee et al. [48]	Mirtazapine on titrated dose (102)	Placebo (102)	P	Y (Secondary)	3	Baseline, week 6, week 12	Dementia Quality of Life ‐ proxy (DEMQOL)	N	Y
Liu et al. [49]	Group music session with percussion instruments (25)	Rest and reading session (25)	NP	Y (Primary)	3	Baseline, week 6, week 12	Hamilton Anxiety Rating Scale (HAM‐A)	N	N
Akhgarjand et al. [50]	Lactobacillus rhamnosus (30)	‐Bifidobacterium longum (30)‐ placebo (30)	P	Y (Secondary)	2	Baseline, week 12	Generalised Anxiety Disorder 7 (GAD‐7)	Y	N
Wu et al. [51]	Accelerated intermittent theta‐burst stimulation (iTBS) (25)	Placebo coil magnetism (24)	P	Y (Secondary)	3	Baseline, week 2, week 10	Hamilton Anxiety Rating Scale (HAM‐A)	N	N
Menengi et al. [52]	Cognitive dual task via telerehabilitation session (10)	Control (no treatment) (10)	NP	Y (Secondary)	2	Baseline, week 6	Beck Anxiety Inventory (BAI)	Y	N
Bhowmik et al. [53]	Adjunct cognitive simulation therapy session (50)	Placebo (50)	NP	Y (Secondary)	2	Baseline, month 6	Neuropsychiatric Inventory Questionnaire (NPI‐Q)	N	N
Monteiro et al. [54]	Intravenous semorinemab (136)	Placebo (136)	P	N	0	Identified as an adverse event	Adverse event only	—	N
Jung et al. [55]	Internet‐based cognitive intervention programme and pharmacological treatment (13)	‐In‐person based cognitive intervention programme and pharmacological treatment (13)‐control ‐ only pharmacological treatment (26)	NP	Y (Primary)	2	Week 0, week 8	Beck Anxiety Inventory (BAI)	Y	N
Atay et al. [56]	Cognitive stimulation therapy (26)	Routine unstructured music, sports, and art activities (26)	NP	Y (Primary)	3	Baseline, post‐treatment, month 3	Geriatric Anxiety Scale (GAS)	Y	N
Howard et al. [57]	Adapted problem adaptation therapy programme (168)	Treatment as usual (168)	NP	Y (Secondary)	4	Baseline, month 3, month 6, month 12	Rating Anxiety in Dementia (RAID)	N	N
Christakou et al. [58]	Combined mental imagery and exercise programme (55)	‐Only exercise programme (52)‐treatment as usual ‐ neither mental imagery nor exercise (53)	NP	Y (Primary)	3	Baseline, week 8, week 12	Short Anxiety Screening Test (SAST)	Y	N
Maleki et al. [59]	Oral pregabalin on titrated dose (28)	Placebo (25)	P	Y (Primary)	3	Baseline, week 4, week 12	Neuropsychiatric Inventory (NPI)	N	N
Fleisher et al. [60]	Intravenous zagotenemab 1400 mg dose (126)	‐Intravenous zagotenemab 5600 mg dose (116)‐placebo (118)	P	N	0	Identified as treatment‐emergent adverse event	Treatment‐emergent adverse event only	—	N
Forstmeier et al. [61]	Cognitive behavioural therapy (20)	Treatment as usual (21)	NP	Y (Secondary)	4	Baseline, post‐treatment, month 6, month 12	Neuropsychiatric Inventory (NPI)	N	N
Dengiz et al. [62]	Mangala strategy game and daily physiotherapy or rehabilitation sessions (20)	Daily physiotherapy or rehabilitation sessions alone (23)	NP	Y (Primary)	2	Baseline post‐ treatment week 6	Hospital Anxiety and Depression Scale (HADS)	Y	N

Anxiety assessment was more common in non‐pharmacological studies (*n* = 17, 62.96%) compared to pharmacological trials (*n* = 10, 37.04%). All 27 studies that assessed anxiety conducted baseline evaluation. The frequency and timing of follow‐up assessments varied considerably across studies, with a mean of 2.6 follow‐up time points per study.

Overall, 12 (44.44%) of the 27 studies used PROMs to evaluate anxiety, while the remaining 15 (55.56%) relied on clinician‐ or informant‐rated tools, including ClinROs and ObsROs completed by informants or caregivers. The Hospital Anxiety and Depression Scale (HADS) was the most commonly used PROM, while the Neuropsychiatric Inventory (NPI) was the most frequently utilised non‐PROM instrument. PROM use was predominantly observed in non‐pharmacological intervention studies.

Overall, only two studies (6.90%) explicitly reported patient and public involvement (PPI); one [41] used a PROM and the other [48] a non‐PROM. Full details of assessment timing, outcome classification, instruments and PPI are presented in Table 3.

### 3.4 Demographic Gaps and Equity Considerations

#### 3.4.1 Sex Differences in Anxiety Measurement

Table 4 presents the sex distribution of participants across the included studies. Female participants constituted the majority in most studies (*n* = 18, 62.07%), while male participants predominated in 11 studies (37.93%). Data on sex were available for a total of 5143 participants with dementia, of whom 2925 (56.87%) were female, and 2218 (43.13%) were male. Two studies (6.90%) employed sex‐specific inclusion criteria: one [49] enrolled only male participants (*n* = 50, 0.80%) and another [35] included only female participants (*n* = 42, 0.17%).

**Table 4:** Sex and racial differences in anxiety measurement.

Study (Author, Year)	Population sample size Enrolled completed (%)	Sex demographics		Ethnicity				
		Male (*n*, %)	Female (*n*, %)	White (*n*, %)	Black (*n*, %)	Asian (*n*, %)	Others (*n*, %)	Under‐reported (*n*, %)
Spector et al. [34]	50 38 (76.00%)	20 (40.00%)	30 (60.00%)	Data not available	1 (2.00%)^c^	Data not available	Data not available	Data not available
Henderson et al. [35]	42 39 (92.86%)	—	42 (100.00%)	41 (97.60%)	1 (2.40%)	0	0	0
Quinn et al. [36]	24 23 (95.83%)	18 (75.00%)	6 (25.00%)	24 (100.00%)	0	0	0	0
Nacu et al. [37]	410 402 (98.05%)	123 (30.60%)^a^	279 (69.40%)^a^	Not reported	Not reported	Not reported	Not reported	Not reported
Pongan et al. [38]	65 59 (90.77%)	20 (33.90%)^a^	39 (66.10%)^a^	Not reported	Not reported	Not reported	Not reported	Not reported
Cheung et al. [39]	165 124 (75.15%)	40 (24.20%)	125 (75.80%)	Not reported	Not reported	Not reported	Not reported	Not reported
Egan et al. [40]	1958 1389 (70.94%)	874 (44.70%)^b^	1083 (55.30%)^b^	1566 (80.00%)^b^	0	342 (17.50%)^b^	35 (1.80%)^b^	14 (0.70%)^b^
Clare et al. [41]	474 426 (89.87%)	248 (52.30%)	226 (47.70%)	457 (96.41%)	7 (1.48%)	6 (1.27%)	4 (0.84%)	0
Zhou et al. [42]	80 78 (97.5%)	33 (41.25%)	47 (58.75%)	Not reported	Not reported	Not reported	Not reported	Not reported
Koch et al. [43]	94 78 (82.98%)	36 (38.00%)	58 (62.00%)	Not reported	Not reported	Not reported	Not reported	Not reported
Maier et al. [44]	110 81 (73.64%)	67 (60.91%)	43 (39.10%)	Not reported	Not reported	Not reported	Not reported	Not reported
Li et al. [45]	90 85 (94.44%)	47 (55.29%)^a^	38 (44.71%)^a^	Not reported	Not reported	Not reported	Not reported	Not reported
Jenewein et al. [46]	54 48 (88.89%)	26 (48.10%)	28 (51.90%)	Not reported	Not reported	Not reported	Not reported	Not reported
Tonga et al. [47]	198 131 (66.17%)	106 (53.50%)	92 (46.50%)	Not reported	Not reported	Not reported	Not reported	Not reported
Banerjee et al. [48]	204 171 (83.82%)	69 (33.80%)	135 (66.20%)	Not reported	Not reported	Not reported	Not reported	Not reported
Liu et al., 2021 [49]	50 50 (100.00%)	50 (100.00%)	—	Not reported	Not reported	Not reported	Not reported	Not reported
Akhgarjand et al. [50]	90 90 (100.00%)	48 (53.33%)	42 (46.70%)	Not reported	Not reported	Not reported	Not reported	Not reported
Wu et al. [51]	49 47 (95.92%)	21 (44.68%)^a^	26 (55.32%)^a^	Not reported	Not reported	Not reported	Not reported	Not reported
Menengi et al. [52]	20 20 (100.00%)	6 (30.00%)	14 (70.00%)	Not reported	Not reported	Not reported	Not reported	Not reported
Bhowmik et al. [53]	100 57 (57.00%)	35 (61.40%)^a^	22 (38.60%)^a^	Not reported	Not reported	Not reported	Not reported	Not reported
Monteiro et al. [54]	272 208 (76.47%)	84 (35.29%)^b^	154 (64.71%)^b^	210 (88.24%)^b^	8 (3.36%)^b^	1 (0.42%)^b^	0	19 (7.98%)^b^
Jung et al. [55]	52 42 (80.77%)	14 (33.33%)	28 (66.70%)	Not reported	Not reported	Not reported	Not reported	Not reported
Atay et al. [56]	52 52 (100.00%)	31 (59.60%)	21 (40.40%)	Not reported	Not reported	Not reported	Not reported	Not reported
Howard et al. [57]	336 279 (83.04%)	131 (39.00%)	205 (61.00%)	318 (94.64%)	5 (1.49%)	5 (1.49%)	8 (2.38%)	0
Christakou et al. [58]	160 142 (88.75%)	43 (26.88%)	117 (73.12%)	Not reported	Not reported	Not reported	Not reported	Not reported
Maleki et al. [59]	54 53 (98.15%)	28 (52.80%)^a^	25 (47.20%)^a^	Not reported	Not reported	Not reported	Not reported	Not reported
Fleisher et al. [60]	360 218 (60.56%)	170 (47.20%)	190 (52.80%)	300 (83.33%)	6 (1.67%)	53 (14.72%)	1 (0.28%)	0
Forstmeier et al. [61]	41 24 (58.44%)	15 (36.60%)	26 (63.40%)	Not reported	Not reported	Not reported	Not reported	Not reported
Dengiz et al. [62]	43 37 (86.05%)	20 (54.10%)^a^	17 (45.90%)^a^	Not reported	Not reported	Not reported	Not reported	Not reported

a Data based on completed sample size.

b Data based on analysed sample size.

c Data based on reported demographic characteristic.

Despite the availability of sex‐disaggregated data, none of the studies using PROMs evaluated whether the validity or effectiveness of these tools differed between sex groups. Additionally, no study stratified anxiety‐related outcomes by sex.

#### 3.4.2 Racial Differences in Anxiety Measurement

Of the 29 included studies, only 8 (27.59%) reported participants’ ethnicity as part of their demographic data, while the remaining 21 studies (72.41%) provided no ethnicity‐related information. Racial and ethnic characteristics reported across the included studies are summarised in Table 4. Among studies reporting ethnicity, data were available for a total of 3432 participants. The majority of the participants identified as White (*n* = 2916, 84.97%), followed by individuals of Asian descent group (*n* = 407, 11.86%). Other ethnic groups were minimally represented, including those classified as Other (*n* = 48, 1.39%), under‐reported/unspecified (*n* = 33, 0.96%) and Black (*n* = 28, 0.82%).

One multi‐centre study [34] reported exclusively Black participants, while another single‐centre study [36] reported an entirely White sample, though this was not protocol‐defined. Both of those studies were conducted in the United Kingdom. None of the included studies that used PROMs examined whether the effectiveness or validity of these tools differed across racial groups. Moreover, no study stratified anxiety‐related outcomes by ethnicity.

## 4 Discussion

The analysis of 29 identified studies involving 5697 participants with dementia revealed inconsistencies in anxiety management approaches, an overrepresentation of Alzheimer's disease, and limited demographic sub‐group analyses.

### 4.1 Importance and Impact of Anxiety in Dementia Population

Anxiety is a prevalent and clinically significant neuropsychiatric symptom in dementia, associated with decreased quality of life and impaired daily functioning [63]. Although anxiety was identified among potential outcome measures in earlier consensus work [64], it was not included in the final Core Outcome Set [25]. Nevertheless, its frequent assessment across included studies, either as a primary, secondary or adverse event outcome, highlights its clinical relevance in dementia research and practice.

Most included studies focused on Alzheimer's disease, which is also the most commonly reported dementia subtype in the global literature [65]. Given that different dementia subtypes may present with distinct clinical trajectories and neuropsychiatric symptom profiles [66, 67], future research should consider broader representation across dementia types to support more comprehensive evidence generation. The mean participant age was 75.7 years, and most studies included individuals with mild or mild‐to‐moderate dementia. This likely reflects the inclusion criteria of the included studies, which predominantly targeted earlier stages of disease where participation in interventions and outcome measurement, including self‐report, is more feasible. As a result, findings may be more representative of earlier‐stage dementia populations. Most studies were conducted in Europe, North America and Asia, and predominantly included White populations, with limited representation from other global regions. This reflects the distribution of the available trials identified in this review. It is important to consider the geographical and ethnic context when interpreting research findings, as healthcare systems, service provision and research infrastructure may differ across settings [68, 69, 70], which may influence the applicability of results in other contexts.

### 4.2 Anxiety Assessment

Heterogeneity in anxiety assessment across studies reflects a lack of standardisation, particularly in the use of dementia‐specific PROMs [11, 18]. Without validated, consistent tools, comparisons across studies are challenging, and patient perspectives may be underrepresented, reducing ecological validity [71, 72].

Most studies assessed anxiety (*n* = 27, 93.1%), yet fewer than half (*n* = 12, 44.44%) used PROMs, mainly in non‐pharmacological interventions. PROMs provide important insight into the subjective experience of anxiety in individuals with sufficient cognitive capacity to self‐report, complementing clinician‐ or observer‐reported measures [73, 74]. Limited PROM use in pharmacological trials may obscure patient‐perceived outcomes [73]. Baseline assessments were universally conducted, but follow‐up timing and frequency varied, potentially affecting comparability. Two studies [54, 60] reported anxiety only as an adverse event, underlining the need for systematic safety monitoring [75, 76].

### 4.3 Anxiety Measurement Tools

Anxiety was measured via PROMs, ClinROs, ObsROs and HRQoL instruments, but tool selection was inconsistent. PROMs capture subjective experiences [73, 74], whereas ClinROs and ObsROs rely on clinician or caregiver observations. Predominant tools included the NPI and HADS. NPI relies on caregiver input, potentially misrepresenting internal states, while HADS may conflate anxiety with depression or cognitive decline [77, 78]. No PROMs were validated specifically for dementia populations, limiting sensitivity and precision [11, 78]. The variability in tools underscores the need for standardised, validated, culturally sensitive instruments to ensure reliable anxiety assessment [79]. Triangulating PROMs with ClinRO (clinician‐reported) and ObsRO (observer‐reported) measures may offer the most comprehensive assessment of anxiety across dementia stages.

### 4.4 Demographic Gaps and Inequities

#### 4.4.1 Sex Differences

Female participants (*n* = 2925, 56.87%) predominated, consistent with national prevalence [65]. Biological factors, longevity and clinical visibility may contribute to this pattern [80, 81]. However, none of the included studies reported sex‐stratified analyses of anxiety outcomes or evaluated measurement properties of PROM by sex. As such, it was not possible within this review to determine whether sex‐specific differences in anxiety measurement or treatment response were examined. Future research may benefit from incorporating sex‐sensitive measures to explore potential differential treatment effects [82].

#### 4.4.2 Racial Differences

Only eight studies reported ethnicity (*n* = 3432), mostly White (*n* = 2916, 84.97%), with minimal representation of Asian, Black or other groups. Limited diversity may restrict generalisability and may obscure potential ethnic differences in dementia risk and treatment response [83, 84, 85]. Improved demographic reporting, inclusive recruitment and culturally sensitive study designs may help address these gaps in future research.

### 4.5 Best Practices in Using Anxiety PROMs

PROMs provide crucial patient‐centred insight into symptom severity, functional impact and quality of life [73, 86]. Despite their value, their use remains inconsistent in dementia trials, partly due to cognitive limitations [19]. Best practice involves multi‐modal assessment combining PROMs with ClinROs and ObsROs, culturally adapted tools, repeated longitudinal administration and integration into shared decision‐making [87, 88]. Standardisation and inclusivity in PROMs are key to improving anxiety measurement, intervention evaluation and equity in dementia research.

### 4.6 Implications for Practice, Policy and Future Research

#### 4.6.1 Practice

Findings from this review highlight inconsistent approaches to anxiety assessment in dementia trials, with limited use of PROMs and a lack of tools validated specifically for dementia populations. In clinical practice, this suggests a need for greater use of validated, dementia‐specific tools where feasible, particularly in mild to moderate dementia where self‐report remains possible. PROMs may support a more patient‐centred understanding of anxiety symptoms and complement clinician‐ and observer‐reported measures. Given the progressive nature of dementia, combining multiple sources of outcome data may provide a more comprehensive assessment of anxiety over time.

#### 4.6.2 Policy

The omission of anxiety from existing core outcome sets, alongside the variability in measurement approaches identified in this review, suggests that emotional outcomes may be underrepresented in standardised dementia outcome frameworks. This may indicate a need to further consider the role of anxiety in future refinements of dementia core outcome sets and clinical guidance. In addition, limited reporting of demographic characteristics highlights the importance of improving standardisation and ensuring culturally and linguistically appropriate measurement approaches.

#### 4.6.3 Research

This review identified a lack of validation of existing tools in dementia populations and limited demographic subgroup analysis. Future research should therefore prioritise the development and validation of anxiety PROMs tailored for people living with dementia, alongside studies examining measurement performance across different stages of disease. Greater inclusion of diverse populations and improved reporting of sex and ethnicity are also needed to support equity in research. Further work is also required to evaluate how PROMs can be integrated into routine clinical workflows, including the potential role of electronic data capture systems.

### 4.7 Implications for Patient and Public Involvement in Outcome Development

Patient and public involvement (PPI) in dementia trials was rarely reported in the included studies [41, 48]. Where reported, development of anxiety PROMs primarily relied on clinician expertise [89] and theoretical [90, 91, 92] or diagnostic frameworks [93, 94], with no explicit involvement of people living with dementia or care partners in item generation or conceptualisation. As a result, it was not possible within this review to determine the extent to which the instruments reflect outcomes that are meaningful from a patient perspective. PROMs are intended to reflect patients’ perspectives and are increasingly recognised as central to improving the quality, relevance and interpretability of research [95, 96]. Current guidance highlights PPI as a key component of high‐quality and person‐centred research and healthcare, contributing to improved outcomes, experiences and decision‐making [95]. Accordingly, PROMs should be co‐developed with patients and the public to ensure they are grounded in lived experience and capture outcomes that matter most [95, 96]. Greater and more consistent involvement of people living with dementia and their care partners across all stages of PROM development may help enhance the validity, applicability and person‐centredness of care outcomes [96, 97].

## 5 Strengths and Limitations

This systematic review has several important strengths. It was prospectively registered on PROSPERO and conducted in accordance with PRISMA guidelines, ensuring methodological transparency and reproducibility. All included studies were randomised controlled trials published within the past decade, providing a contemporary and robust evidence base. The inclusion of both pharmacological and non‐pharmacological interventions allowed for a broad examination of anxiety assessment practices in dementia trials. In addition, the review uniquely focused on anxiety and the use of PROMs, addressing an underexplored yet clinically important aspect of dementia research and highlighting key methodological and equity‐related gaps. Several limitations should be acknowledged. The review was limited to English‐language publications and to a defined publication period (2015–2025), which may have excluded earlier studies and potentially relevant historical evidence. Substantial heterogeneity in study design, interventions and outcome measures precluded meta‐analysis and limited direct comparability across studies. Many trials had small sample sizes, reducing external validity. Reporting of ethnicity was inconsistent, and no studies examined whether anxiety measures performed differently by sex or racial or ethnic background, limiting assessment of equity and generalisability. Furthermore, most studies were conducted in high‐income countries, restricting applicability to low‐ and middle‐income settings.

From a measurement perspective, while validated anxiety tools were widely used, few studies employed PROMs, and none used tools validated specifically for people living with dementia. Reliance on clinician‐ or caregiver‐reported measures may have obscured subjective anxiety experiences, particularly among individuals with mild to moderate dementia who remain capable of self‐report. Although this review identified PROMs used to assess anxiety in people living with dementia, a formal evaluation of measurement properties using the COSMIN framework was beyond the scope of the review. Future research would benefit from a COSMIN‐based appraisal to further evaluate the psychometric quality and suitability of the identified instruments. Despite these limitations, this review provides a comprehensive and critical synthesis of current practices in anxiety assessment within dementia trials and identifies clear priorities for future research, including standardisation of outcome measures, greater use of dementia‐appropriate PROMs and more inclusive and equitable study designs.

## 6 Conclusion

This systematic review demonstrates considerable heterogeneity in the assessment of anxiety in dementia clinical trials, including variation in outcome measures, assessment timing and demographic reporting. Although anxiety was frequently measured, fewer than half of studies used PROMs, and none employed tools validated specifically for dementia populations [18]. This limits comparability across studies and constrains the integration of patient‐centred outcomes in dementia research. Inconsistent reporting of sex and ethnicity, combined with the predominance of studies from high‐income countries, further reduces the generalisability of findings. Future research should prioritise the validation and routine use of dementia‐appropriate, culturally sensitive anxiety PROMs and promote their inclusion within dementia core outcome sets to support equitable, person‐centred outcome assessment.

## Author Contributions

**Jason Domingo:** conceptualization, methodology, investigation, validation, formal analysis, data curation, software, visualization, writing – original draft, writing – review and editing, supervision, project administration, resources, funding acquisition. **Ana Alves:** investigation, validation. **Julie Sanders:** conceptualization, methodology, formal analysis, writing – review and editing, supervision, project administration, resources.

## Funding

The authors have nothing to report.

## Ethics Statement

As this study is a systematic review of existing literature, ethical approval from an Ethics or Trust committees was not required.

## Conflicts of Interest

The authors declare no conflicts of interest.

## Supporting information

Supporting File

## Data Availability

The data that support the findings of this study are available from the corresponding author upon reasonable request.
