# Supplementary figures and images for: Evaluating the Use of Anxiety Patient‐Reported Outcome Measures (PROMs) in Dementia Clinical Trials: A Systematic Review
Source: Health Expect. 2026 Jun 21;29(3):e70736. doi: 10.1111/hex.70736 (PMC13283353; doi:10.1111/hex.70736)

**Supplementary Material**

**
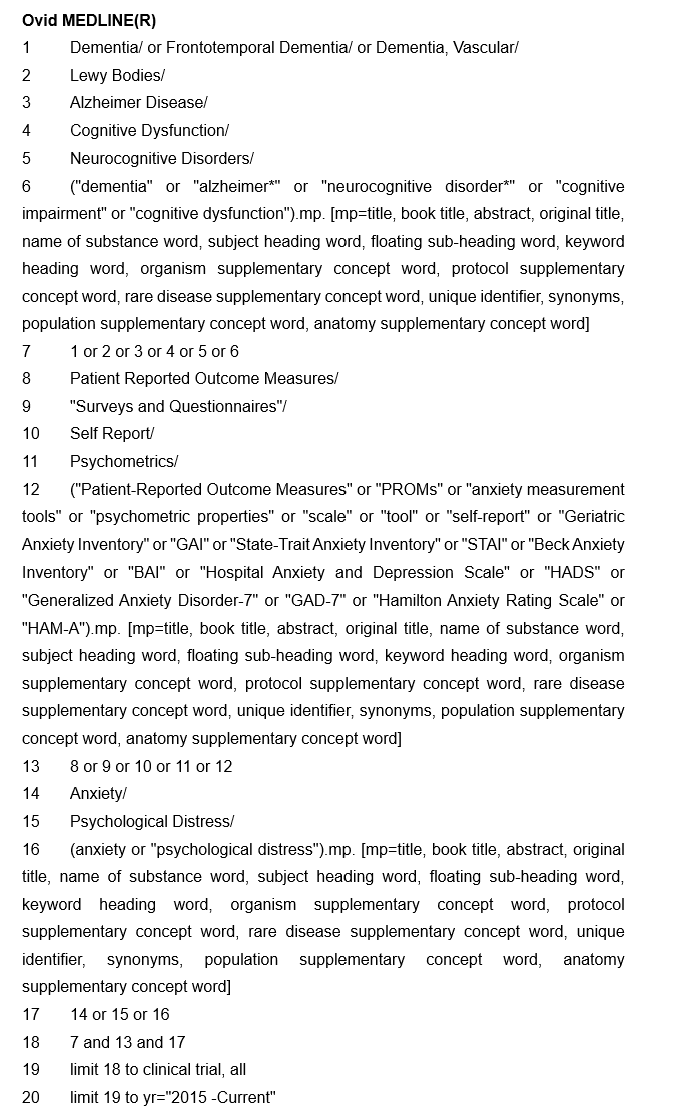
**

**
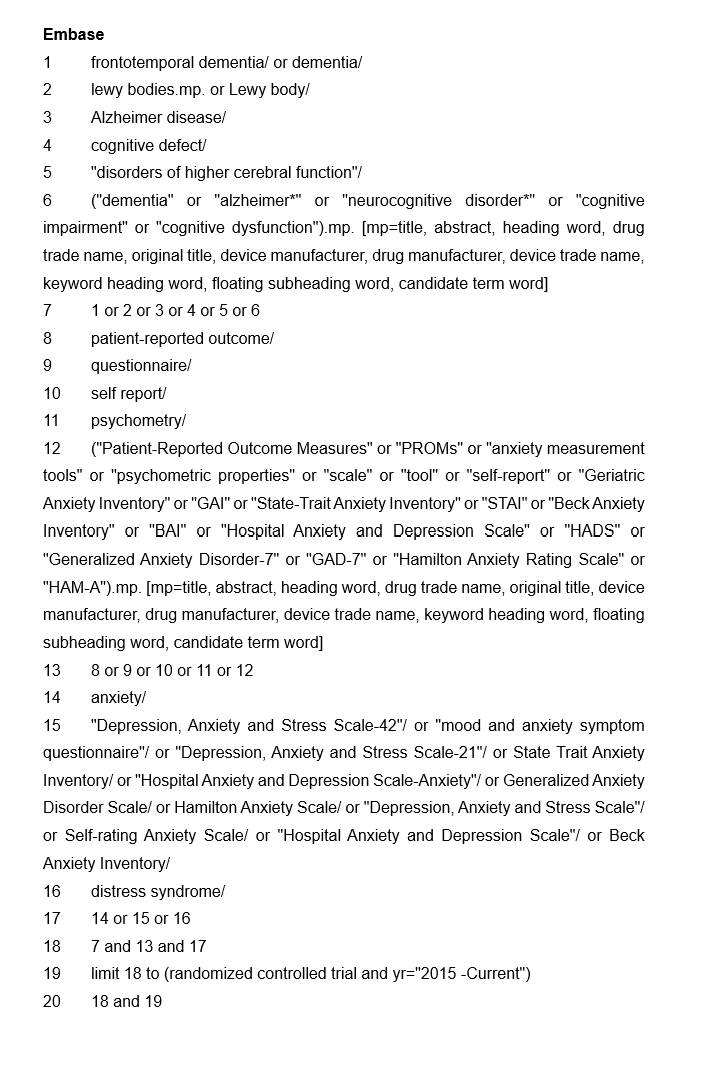
**

**
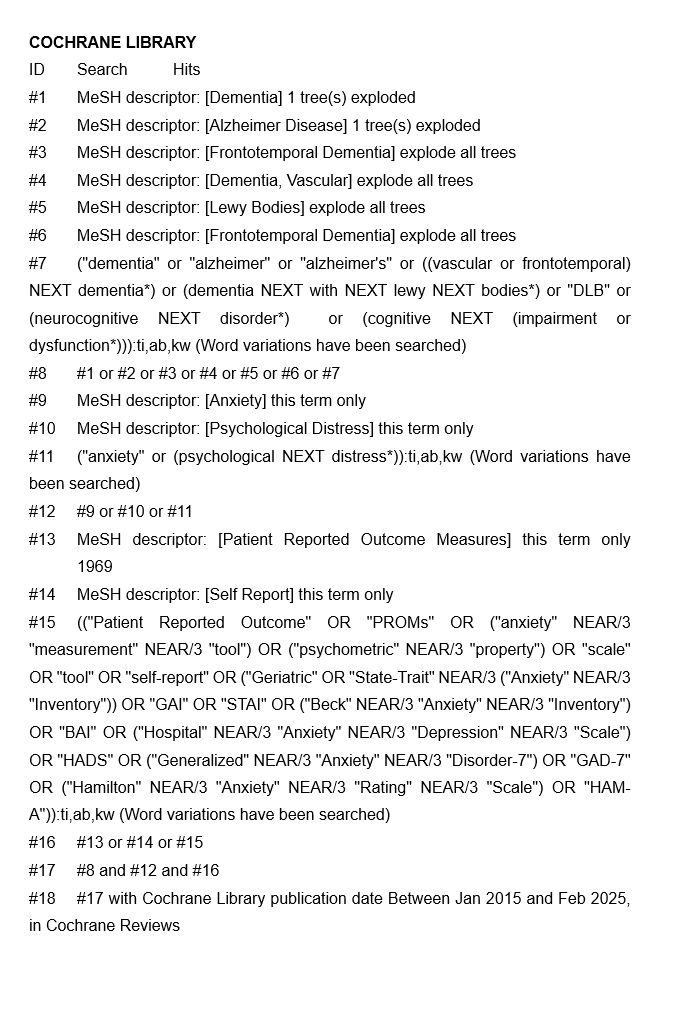
**

**
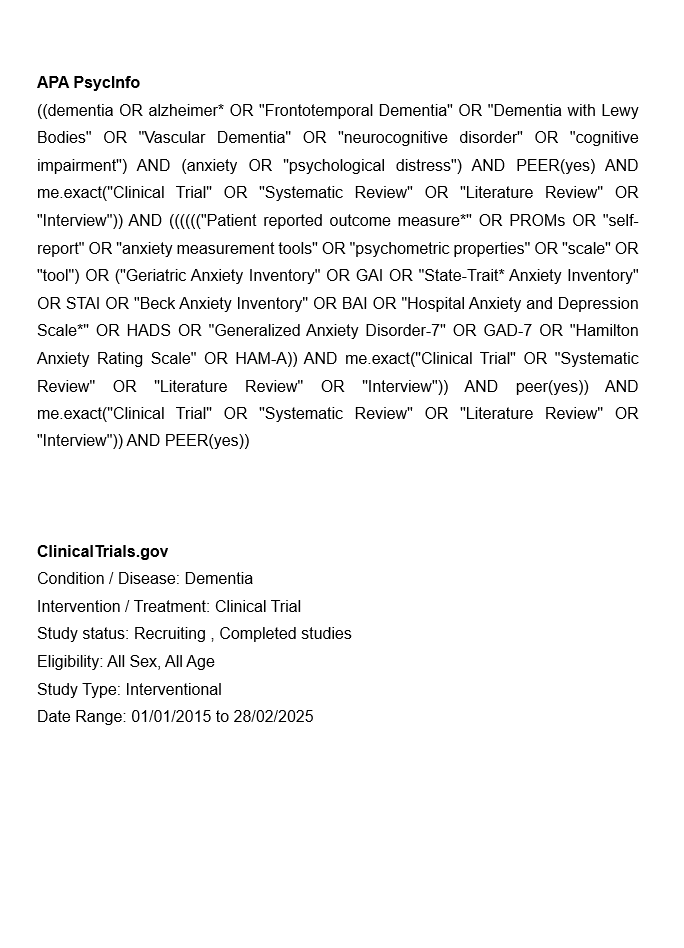
**

Supplement: Supplementary file 1 — Supporting File [file HEX-29-e70736-s001.docx]
